# Expression and significance of transforming growth factor-β receptor type II and DPC4/Smad4 in non-small cell lung cancer

**DOI:** 10.3892/etm.2014.2065

**Published:** 2014-11-12

**Authors:** HONG CHEN, JING-WEI WANG, LI-XIN LIU, JI-DONG YAN, SHU-HUA REN, YAN LI, ZHENG LU

**Affiliations:** 1 Department of Radiotherapy and Chemotherapy, Tangshan Gongren Hospital, Tangshan, Hebei 063000, P.R. China; 2 Department of Thoracic Surgery, Tangshan Gongren Hospital, Tangshan, Hebei 063000, P.R. China

**Keywords:** transforming growth factor-β receptor type II, DPC4/Smad4, non-small cell lung cancer

## Abstract

The aim of the present study was to investigate the expression levels of transforming growth factor-β (TGF-β) receptor type II (TβRII) and DPC4/Smad4 in the TGF-β signaling pathway and the importance of these expression levels in non-small cell lung cancer (NSCLC). The mRNA and protein expression levels of TβRII and DPC4/Smad4 were detected by reverse transcription-quantitative polymerase chain reaction and western blotting, respectively, in NSCLC and control nonlesional lung tissues of 60 patients. The protein expression levels of DPC4/Smad4 were detected by immunohistochemistry in paraffin-embedded samples of NSCLC. In addition, the correlations among the expression levels of TβRII and DPC4/Smad4 and their association with the clinical and pathological features of NSCLC were analyzed. The expression levels of TβRII and DPC4/Smad4 in NSCLC tissues were significantly lower when compared with the control nonlesional lung tissues (P<0.05). In addition, the expression of TβRII and DPC4/Smad4 in poorly-differentiated NSCLC tissues was significantly lower compared with moderately- or well-differentiated NSCLC tissues (P<0.05). The expression levels of TβRII and DPC4/Smad4 were significantly lower in NSCLC tissues with metastatic lymph nodes compared with tissue without metastatic lymph nodes (P<0.05). Thus, the expression levels were demonstrated to significantly correlate with the clinical and pathological stages, and subsequently were shown to be associated with the occurrence and progression of NSCLC. In conclusion, TβRII and DPC4/Smad4 may play an important role in the tumorigenesis, differentiation and progression of NSCLC via the TGF-β signaling pathway.

## Introduction

Non-small cell lung cancer (NSCLC) is the leading cause of cancer-related mortality worldwide, accounting for 80–85% of lung cancer cases (1). Transforming growth factor-β (TGF-β) plays a critical role in regulating the proliferation, differentiation and apoptosis of cells, as well as the development of embryos. The TGF-β family includes several isoforms (TGF-β 1, 2 and 3), which interact with the specific cellular serine/threonine kinase receptors, TGF-β receptor type I (TβRI) and type II (TβRII) (2). The heteromeric complexes of these receptors activate Smad proteins in order to regulate the expression of target genes. Among the members of the Smad family, Smad4 is particularly associated with cancer (3). Hahn *et al* (4) identified that a TβRII and/or Smad4 gene deletion, point mutation or functional inactivation occurs in a variety of tumors.

At present, there is increasing evidence that impaired signal transduction is closely associated with the occurrence of tumors. TGF-β/Smad is one of two major pathways for adjusting cell proliferation; TGF-β and Smad may work together and contribute to the expression of specific genes (5). Smad4 deletion or mutation can induce precancerous diseases, promoting tumor development and affecting the biological behavior of these tumors, such as tumor invasion and metastasis (5). However, studies on the expression of TβRs and DPC4/Smad4 in NSCLC are limited. Takanami *et al* (6) found that the presence of immunoreactivity for TβRI and/or TβRII is correlated with poor prognosis in lung adenocarcinoma. In the present study, the mRNA and protein expression levels of TβRII and DPC4/Smad4 were compared between paired samples of NSCLC and nonlesional lung tissues using reverse transcription-quantitative polymerase chain reaction (RT-qPCR), western blotting and a quantitative immunohistochemistry method. In addition, the associations with clinical and pathological features of NSCLC were analyzed.

## Materials and methods

### Patients

Lung tumor tissue specimens were obtained from 60 patients (male, 40; female, 20) that had undergone a lobectomy and mediastinal lymph node dissection for primary lung tumors at the Department of Thoracic Surgery at the Tangshan Gongren Hospital (Tangshan, China) between January 2008 and December 2009. Control nonlesional lung tissue specimens from areas distal to the tumor were obtained from the same patients (n=24). None of the patients had received preoperative radiotherapy or chemotherapy. The mean age of the patients was 55.62 years (range, 33–78 years). The types of tumors identified were squamous cell carcinoma (n=27), adenocarcinoma (n=23), large cell carcinoma (n=3) and adenocarcinoma-squamous cell carcinoma (n=7), which were histologically graded as well- (n=18), moderately- (n=20) and poorly-differentiated (n=22). In addition, lymph node metastasis was diagnosed in 33 patients. The patients were classified into clinical stages I (n=16), II (n=24) and III (n=20), according to the TNM staging system (7). Partial tumors and control nonlesional lung tissues were obtained during surgery, frozen immediately with liquid nitrogen and stored in a freezer at −70°C.

Furthermore, 60 paraffin-embedded specimens obtained between 2000 and 2008, along with the five-year follow-up data, were used in an additional investigation, which included 60 patients (male, 30; female, 30; age range, 30–74 years). All the specimens underwent pathological analysis to determine the degree of differentiation, histology and clinical staging. The study was conducted in accordance with the Declaration of Helsinki and was approved by the Ethics Committee of Tangshan Gongren Hospital. Written informed consent was obtained from all the participants.

### RT-qPCR

To measure the mRNA expression levels of TβRII and DPC4/Smad4, an RT-qPCR method was employed. Total RNA was extracted from the tumor and control nonlesional lung tissues using TRIzol reagent (Invitrogen Life Technologies, Carlsbad, CA, USA), according to the manufacturer’s instructions. The tissue was homogenized in 1 ml TRIzol to isolate the total RNA (2 μg), which was reverse transcribed into cDNA. The primers used were as follows: TβRII sense, 5′-GGG AAC AAC ATG CTA AAT GG-3′ and antisense, 5′-CTG CAA CCA GAA CCT CAA GT-3′; β-actin sense, 5′-ACC ACA GTC CAT GCC ATC AC-3′ and antisense, 5′-TCC ACC ACC CTG TTG CTG TA-3′; Smad4 sense, 5′-AAAGGTGAAGGTGATGTTTGGGTC-3′ and antisense, 5′-CTGGAGCTATTCCACCTACTGATCC-3′; β-actin sense, 5′-CCACCCATGGCAAATTCCATGGCA-3′ and antisense, 5′-TCAAGACGGCAGGTCAGGTCCACC-3′. The primers were annealed at 58°C for 28 cycles, and each sample was reverse transcribed in duplicate. To quantify the expression of the target gene, 10-μl samples of the PCR products were separated electrophoretically on a 1.5% agarose gel. The expression of β-actin was used as an internal control and the products were semi-quantified by Gel-Pro Analyzer image analysis software (Media Cybernetics, Inc., Silver Spring, MD, USA).

### Western blotting

Lung cancer and control nonlesional lung tissues were treated with radioimmunoprecipitation assay buffer and incubated on ice. The protein concentration was determined with the Coomassie Brilliant Blue method. To perform polyacrylamide gel electrophoresis, each lane of the gel was loaded with 50 g protein mixture, and transferred to a nylon membrane. Hybridization occurred following the addition of primary rabbit-anti-human antibodies against TβRII and DPC4/Smad4 (1:500; Santa Cruz Biotechnology, Inc., Santa Cruz, CA, USA) and a secondary goat-anti-rabbit antibody (1:5,000; Santa Cruz Biotechnology, Inc.). Color reaction and exposure in a dark room were performed to develop the films. Band Leader software was used to analyze the ratio of proteins to β-actin (internal control).

### Immunohistochemistry

The specimens were deparaffinized in xylene for 20 min, and rehydrated with graded ethanol solutions. The endogenous peroxidase was blocked by incubating the sections in 3% hydrogen peroxide and methanol for 15 min. Antigen retrieval was performed by heating the deparaffinized sections at 121°C for 10 min in l0 mmol/l citrate buffer solution (pH 6.0) in an autoclave. After blocking nonspecific reactivity with 10% normal goat serum for 10 min at room temperature, the specimens were incubated overnight at 4°C with a primary antibody against Smad4 (1:100), followed by 30 min incubation at 37°C with a secondary antibody (goat-anti-rabbit; 1:l,000). The samples were subsequently treated with the streptavidin biotin complex. Staining of the specimens was performed using 3,3′-diaminobenzidine, followed by counterstaining with hematoxylin, dehydration and cover-slipping with mounting medium. The presence of brown yellow particles in the cells following the immunohistochemical assay indicated positively-stained cells. The degree of staining was determined based on the percentage of positive cells, and the specimens were labeled with (−) if they contained <5% positive cells, (+) for 5–20% positive cells and (++) for >20% positive cells. Positive expression was recorded in the specimens labeled as (++) following immunohistochemistry.

### Statistical analysis

Statistical analysis was done using the SPSS statistical software (SPSS, Chicago, IL, USA). The difference between TβRII and DPC4/Smad4 expression in tumor tissues and normal tissues was performed by unpaired Student’s t test. The correlation between TβRII and Smad4 expression and the clinicopathological characteristics were analyzed using the Chi-squared test and Spearman’s correlation analysis. P<0.05 was considered to indicate a statistically significant difference.

## Results

### mRNA and protein expression levels of TβRII

**Figure 1:**
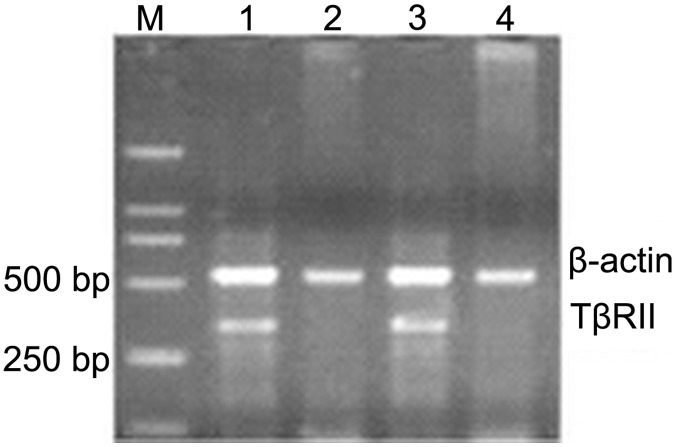
Agarose gel electrophoresis image demonstrating the mRNA expression of TβRII in control nonlesional lung (lanes 1 and 3) and non-small cell lung cancer tissues (lanes 2 and 4). β-actin was used as the internal control. TβRII, transforming growth factor-β receptor type II.

**Figure 2:**
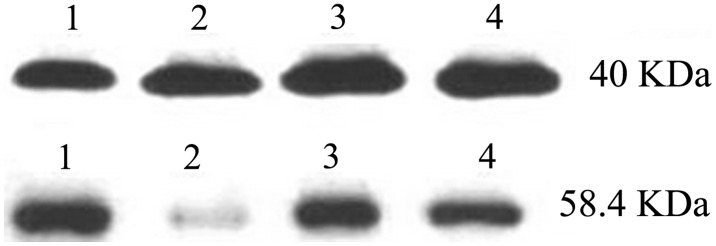
Protein expression of transforming growth factor-β receptor type II in control nonlesional lung (lanes 1 and 3) and non-small cell lung cancer tissues (lanes 2 and 4).

RT-qPCR analysis demonstrated that the relative expression of TβRII in NSCLC tissues was 0.498±0.198, which was markedly lower compared with the control nonlesional lung tissues (1.820±0.672; P<0.05; Fig. 1). Similarly, the western blotting results demonstrated that the relative expression of TβRII was 0.203±0.142 in the NSCLC tissues and 0.882±0.334 in the control nonlesional lung tissues, revealing a statistically significant difference (P<0.05; Fig. 2). β-actin (40 kDa) was used as an internal control.

### Correlation between TβRII and Smad4 protein expression

**Figure 3:**
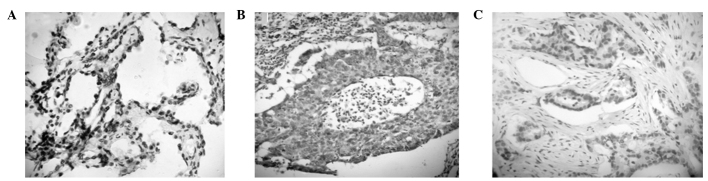
Protein expression of transforming growth factor-β receptor type II and Smad4, as detected using an immunohistochemical assay. (A) Positive expression of Smad4 protein in control nonlesional lung tissues. Negative expression of Smad4 protein in (B) lung squamous carcinoma and (C) lung adenocarcinoma.

Protein expression levels of TβRII and Smad4 were investigated. An immunohistochemical assay revealed that Smad4 was mainly expressed in the cell nucleus of NSCLC and control nonlesional lung tissues. Positive expression of both TβRII and Smad4 was identified in eight NSCLC tissue samples, while negative expression of the two proteins was identified in 36 cases (Fig. 3). Correlation analysis indicated that the expression of Smad4 was positively associated with the expression of TβRII in NSCLC tissues (r=0.2326, P<0.01).

### Correlation between TβRII and Smad4 expression with clinical pathology parameters

The positive expression rate of TβRII and Smad4 in the poorly-differentiated lung carcinoma group was significantly lower when compared with the well- and moderately-differentiated lung carcinoma groups, exhibiting a statistically significant difference (P<0.05). In addition, the expression levels in the patients with lymph node metastasis were significantly lower when compared with the patients without lymph node metastasis, and a statistically significant difference was observed between the two groups (P<0.05). The positive expression rate of TβRII and Smad4 was also reduced in the patients with higher tumor stages. Moreover, a statistically significant difference was observed between the poorly-differentiated and the well- and moderately-differentiated groups (P<0.05; Table I).

## Discussion

Smad4 was identified as a candidate tumor suppressor gene in pancreatic carcinomas and was initially known as ‘deleted in pancreatic carcinoma locus 4 (DPC4)’, since almost 40% of patients had a deleted or inactivated version of Smad4 (4). The Smad4 protein is a critical transcription factor in the TGF-β signaling pathways. Ke *et al* (8) indicated that DPC4 may be involved in preventing tumor metastasis by inhibiting tumor angiogenesis.

Decreased expression of TβR was considered to be one of the mechanisms underlying the loss of TGF-β sensitivity and the enhanced tumor progression in numerous types of cancer (9–11). Decreased mRNA and corresponding protein expression levels of TβRII have been reported in gastric cancer cell lines (12). However, studies on the expression of TβR in NSCLC have been rarely reported (6,13,14). In the present study, RT-qPCR, western blotting and immunohistochemistry were performed to analyze the mRNA and immunoreactive protein expression levels of TβRII and Smad4. The aim of the study was to compare the mRNA and protein expression levels of TβRII and Smad4 in NSCLC and control nonlesional lung tissues.

The present study demonstrated that immunoreactive Smad4 protein is expressed significantly less in NSCLC tissues compared with control nonlesional lung tissues, and a similar trend is present for the mRNA expression levels. The mRNA expression of Smad4 in poorly-differentiated NSCLC tissues was significantly lower compared with moderately- or well-differentiated NSCLC tissues (P<0.05). In addition, the mRNA expression levels of Smad4 were significantly lower in NSCLC tissues with metastatic lymph nodes compared with tissues without metastatic lymph nodes (P<0.05). Protein expression was found to be significantly decreased in cancer tissues, and the expression was demonstrated to be closely associated with higher clinical staging, the presence of metastatic lymph nodes and poor differentiation. In addition, a decrease in the mRNA expression of Smad4 was found to be associated with a decrease in the protein expression of Smad4. The results indicated that the expression of Smad4 was associated with the tumorigenesis, differentiation and progression of NSCLC. Previous studies have indicated that patients with Smad4-positive tumors have a longer survival rate compared with patients without Smad4-labeled tumors (15,16).

TGF-β is a multifunctional cytokine that inhibits epithelial cell proliferation, and a strong correlation has been demonstrated between malignant progression and loss of sensitivity to the antiproliferative effects of TGF-β. Tumor cells often escape the antiproliferative effects of TGF-β by the mutational inactivation or dysregulated expression of components in the TGF-β signaling pathway (17). A decreased expression of TβRs is considered to be a possible mechanism underlying the loss of TGF-β sensitivity and the enhanced tumor progression in numerous types of cancer (9–11). Accordingly, the loss of growth regulation by TGF-β is considered to be an important step in tumor progression in several types of cancer (18–20).

Decreased mRNA and corresponding protein expression of TβRII has been reported in a number of tumor cell lines (14,16,18,19). In the present study, the mRNA and protein expression levels of TβRII were further investigated in NSCLC and control nonlesional lung tissues, revealing that the expression levels in NSCLC tissues were lower compared with the control nonlesional lung tissues. Positive expression rates of TβRII in the poorly-differentiated and lymph node metastasis groups were significantly lower compared with the well-differentiated and no lymph node metastasis groups, respectively, and the differences were found to be statistically significant (P<0.05). The positive expression of TβRII decreased with increasing pathological stage. In addition, the present study further demonstrated that the protein expression levels of TβRII and Smad4 in NSCLC were positively correlated, indicating that TβRII and Smad4 proteins may have a synergistic effect in the development of NSCLC.

In conclusion, the present study demonstrated that downregulation of Smad4 gene expression may be involved in lung carcinogenesis. The results indicated that loss of the growth inhibitory response to TGF-β signaling may be crucial in promoting tumor development in NSCLC.

## Figures and Tables

**Table I:** Associations between the expression of TβRII and DPC4/Smad4 and clinical pathology in patients with NSCLC.

Clinical pathology parameter	Patients (n)	TβRII (n)		χ^2^	P-value	DPC4/Smad4 (n)		χ^2^	P-value
	
		+	−			+	−		
Histological grade									
Well- and moderately-differentiated	38	24	14			20	13		
Poorly-differentiated	22	6	16	7.17	<0.05	4	18	9.15	<0.05
Lymph node metastasis									
No metastasis	27	17	10			16	11		
Metastasis	33	7	26	10.79	<0.05	10	23	5.07	<0.05
Clinical stage									
I+II	40	24	16			22	18		
III	20	4	16	8.57	<0.05	3	17	8.78	<0.05

NSCLC, non-small cell lung cancer; TβRII, transforming growth factor-β receptor type II.
